# Inflammation Induces Irreversible Biophysical Changes in Isolated Nucleus Pulposus Cells

**DOI:** 10.1371/journal.pone.0099621

**Published:** 2014-06-17

**Authors:** Robert Maidhof, Timothy Jacobsen, Angelos Papatheodorou, Nadeen O. Chahine

**Affiliations:** 1 Center for Autoimmune and Musculoskeletal Diseases, The Feinstein Institute for Medical Research, North Shore-LIJ Health System, Manhasset, New York, United States of America; 2 Hofstra-North Shore LIJ School of Medicine, Hempstead, New York, United States of America; National Centre for Scientific Research, ‘Demokritos’, Greece

## Abstract

Intervertebral disc degeneration is accompanied by elevated levels of inflammatory cytokines that have been implicated in disease etiology and matrix degradation. While the effects of inflammatory stimulation on disc cell metabolism have been well-studied, their effects on cell biophysical properties have not been investigated. The hypothesis of this study is that inflammatory stimulation alters the biomechanical properties of isolated disc cells and volume responses to step osmotic loading. Cells from the nucleus pulposus (NP) of bovine discs were isolated and treated with either lipopolysaccharide (LPS), an inflammatory ligand, or with the recombinant cytokine TNF-α for 24 hours. We measured cellular volume regulation responses to osmotic loading either immediately after stimulation or after a 1 week recovery period from the inflammatory stimuli. Cells from each group were tested under step osmotic loading and the transient volume-response was captured via time-lapse microscopy. Volume-responses were analyzed using mixture theory framework to investigate two biomechanical properties of the cell, the intracellular water content and the hydraulic permeability. Intracellular water content did not vary between treatment groups, but hydraulic permeability increased significantly with inflammatory treatment. In the 1 week recovery group, hydraulic permeability remained elevated relative to the untreated recovery control. Cell radius was also significantly increased both after 24 hours of treatment and after 1 week recovery. A significant linear correlation was observed between hydraulic permeability and cell radius in untreated cells at 24 hours and at 1-week recovery, though not in the inflammatory stimulated groups at either time point. This loss of correlation between cell size and hydraulic permeability suggests that regulation of volume change is disrupted irreversibly due to inflammatory stimulation. Inflammatory treated cells exhibited altered F-actin cytoskeleton expression relative to untreated cells. We also found a significant decrease in the expression of aquaporin-1, the predominant water channel in disc NP cells, with inflammatory stimulation. To our knowledge, this is the first study providing evidence that inflammatory stimulation directly alters the mechanobiology of NP cells. The cellular biophysical changes observed in this study are coincident with documented changes in the extracellular matrix induced by inflammation, and may be important in disease etiology.

## Introduction

The nucleus pulposus (NP) is the central region of the disc that is comprised of cells that maintain a matrix rich in proteoglycans and a high water content [1], [2]. NP cells are subjected to biophysical forces including hydrostatic stress and osmotic pressure as the vertebral bodies impart axial loading on the disc. These biophysical factors are known to regulate NP cell volume [3], gene expression [4], [5] and protein synthesis [6], [7] during development [8], homeostasis [2], and in disc disease [9].

Disc degeneration (DD) is characterized by changes in extracellular matrix (ECM) properties including loss of proteoglycans and collagens, degenerative fibrillation, and decreased water content [10], [11], [12], which alter the disc’s ability to bear load. These pathophysiological changes can result in decreased osmotic pressure [13], [14] that can further impact cell mechanobiology.

Degenerate discs exhibit higher levels of pro-inflammatory cytokines, such as TNF-α, IL-1β, and others relative to non-degenerate discs [15], [16], [17], [18], thus implicating inflammation as a mediator of the degenerative cascade. Disc cells respond to TNF-α and IL-1β stimulation by down regulating synthesis of matrix proteins and increasing expression of matrix-degrading enzymes, leading to net catabolism [19], [20], [21], [22]. Recently we have shown that activation of the toll-like receptor 4 (TLR4) pathway in disc cells *in vitro* with the trigger ligand lipopolysaccharide (LPS) upregulates a cascade of pro-inflammatory cytokines [23]. Furthermore, injection of LPS into the disc *in vivo* leads to increased matrix fibrillation, decreased cellularity, and loss of compressive stiffness [23]. Therefore inflammatory activation alone may be sufficient to provoke the biochemical and biomechanical changes associated with DD.

While inflammatory signaling affects NP cell metabolism by altering molecular expression patterns favoring matrix catabolism, it is unknown if inflammation directly alters the biophysical properties of cells. In this study we test the hypothesis that inflammatory stimulation alters the biomechanical properties of NP cells and volume responses to step osmotic loading. We treat NP cells with an inflammatory stimulus (LPS or TNF-α) for 24 hours and measure cellular volume regulation responses to osmotic loading either immediately after stimulation or after a one week recovery period from the inflammatory stimuli. At both experimental time points, we measure the volume-response of isolated NP cells to osmotic loading using a custom microfluidic chamber [24] and analyze this response using a mixture theory model [25], [26], [27] yielding two cell biophysical properties, hydraulic permeability and intracellular water content. To identify potential mechanisms by which NP cells may alter their biomechanical properties in response to osmotic loading we also analyzed alterations in cell cytoskeletal structure and water channel (aquaporin) expression. We found that inflammatory treatment causes significant changes in NP cell biophysical properties and these changes are not reversible after 1 week recovery post inflammatory stimulation.

## Methods

### Cell Isolation and Culture

NP tissue was harvested under sterile conditions from lumbar disc segments of freshly slaughtered juvenile cows obtained from an abattoir (Green Village Packing Company, Green Village, NJ, USA; permission was obtained to use these animal parts for research). NP cells were isolated from tissue via enzymatic digestion and cultured in complete media (high glucose DMEM+10% FBS+1% antibiotic/antimycotic). Cells were expanded (P2–4) and cultured (5×10^4^ cells/cm^2^) in complete media for 1 day prior to inflammatory treatment.

### Inflammatory Treatment

LPS (from *Escherichia coli* 0111: B4, Sigma-Aldrich, St. Louis, MO, USA) was suspended in sterile dH_2_O by sonication, diluted in serum-free media, and sonicated again immediately prior to use. Recombinant rat TNF-α (R&D Systems, Minneapolis, MN, USA) was suspended in sterile dH_2_O with 0.1% bovine serum albumin, diluted in serum-free media and sonicated prior to use. Cells were rinsed in PBS and cultured in either serum-free DMEM (untreated), DMEM containing 1 µg/ml LPS (LPS), or DMEM containing 10 ng/ml TNF-α (TNF-α) for 24 hours. No significant changes in culture media osmolarity were observed as a result of LPS or TNF-α addition (untreated: 341.7±0.6 mOsm/L, LPS: 340.3±0.6 mOsm/L, TNF-α: 342±1.0 mOsm/L, p = 0.07), as measured with a freezing point osmometer (Advanced Instruments, Norwood, MA, USA). Cells were analyzed immediately or rinsed with PBS and returned to serum-containing growth media without inflammatory stimulants for an additional one week of culture (untreated recovery, LPS recovery, and TNF-α recovery) with one media change on day 4 in culture.

### Osmotic Loading and Imaging

NP cells were seeded in a custom-made Y-shaped microfluidic channel as previously described [24], [27]. The polydimethylsiloxane (PDMS) channel (1 cm long, 300 µm width, 100 µm height) was sealed over a poly-D-lysine treated glass slide with two open reservoirs at the upstream end and one reservoir at the downstream end. For each test, NP cells were introduced to the channel through addition of 40 µl of cell suspension. Cells seeded in the channels were incubated at 37°C for 30 min to initiate adhesion but retain a primarily rounded morphology. In preliminary trials, seeding times greater than 1 hour resulted in predominantly flattened cells. However, we chose to test spherical cells because NPs assume a round morphology *in vivo* and this conformation allowed us to estimate cell volume from cross-sectional area measurements.

Flow was introduced through a hydrostatic pressure gradient by the addition of 100 µL of 333 mOsm/L NaCl at to each of the upstream reservoirs. After a 5 minute cell equilibration period, the up- and down-stream reservoirs were emptied and flow was re-established with the next NaCl test solution. Loading was performed at room temperature to observe passive osmotic properties since it has been previously shown that cells do not exhibit active volume recovery when loaded with NaCl under this condition [27]. In each experiment osmotic loading was applied in a sequence of decreasing osmolarity steps of 600, 466, 333, and then 200 mOsm/L NaCl. This range was selected based on typical osmotic concentrations found within the healthy and degenerated disc [14] and to explore the theory previously verified for articular chondrocytes [27].

During the transient osmotic steps, cells were imaged through the glass slide with an inverted microscope (Fluoview 300, Olympus, Center Valley, PA, USA) with differential interference contrast (DIC) filter and 20x objective focused on the cells’ midplane. Images (512×512 pixels, 0.20 µm/pixel) were acquired at regular time intervals (0.5 Hz). A custom Matlab routine was used to segment individual cells in each image based on a previously published algorithm [28] and calculate the volume response over time for each cell under the assumption of spherical geometry (n = 7–12 cells per group).

Cell radius measurements were performed on cells plated in the microfluidic channels for 30 min, loaded with 333 mOsm/L NaCl, imaged via inverted microscope, and segmented using the Matlab routine. Due to the smaller effect size in the radii measurements, a greater number of observations were made (n = 52–127 cells per group) compared to the volume-response tests (n = 7–12 cells per group). Separate cell preparations were used to perform transient osmotic studies and cell size studies.

### Analysis of Volume Response

The volume response of cells to osmotic loading was analyzed with the Kedem-Kachalsky model, formulated using equations based on a mixture theory framework [25], [26], [27] (n = 7–12 cells per osmotic step). NaCl was modeled as a non-permeating solute at room temperature [27], [29]. Therefore, the differential equation governing the cell volume 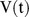 simplifies to
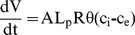
(1)where A is the volume-dependent cell surface area (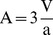, 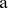 is the cell radius), 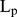 is the membrane hydraulic permeability, 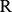 is the universal gas constant, 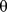 is the absolute temperature, and 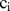 and 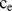 are the intracellular and extracellular osmolarities, respectively. Experimentally, the extracellular osmolarity was defined by step application of NaCl solutions. The intracellular osmolarity is related to the number of moles of solute within the cell (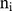) and the cell volume by
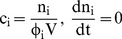
(2)where 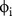 is the volume fraction of osmotically active water in the cytoplasm. As the cell expands, this volume fraction changes as water enters the cell according to the principle of conservation of mass
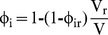
(3)where the subscript r denotes the reference state (prior to each loading step). The reference intracellular water content (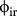) can be deduced from these equations in the limit when 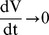 (i.e. after the cell reaches equilibrium volume) from the equation 
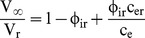
(4)


The measured cell volume response was curve fit to these equations using a custom Matlab routine to calculate the parameters 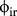 and 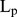 for each cell at each osmotic loading step.

### Nitric Oxide Assay

Cells were plated in 96-well plates and exposed to LPS, TNF-α, or control conditions for 24 hours. Supernatants were analyzed for nitrite, a breakdown product of released nitric oxide (NO), according to manufacturer’s protocols (Griess Reagent Kit, Promega, Madison, WI, USA). The wells were then rinsed with PBS and returned to growth media for an additional one week, with a media change at experimental day 4. Supernatants were again analyzed for nitrite at days 4 and 7 of culture. Total nitrite release was computed as the cumulative release measured at each of the 3 time points. Two independent experiments were performed with n = 6 wells per experiment.

### Gene Expression

Gene expression levels were determined by quantitative RT-PCR. NPs were cultured in 6-well plates (9.5 cm^2^ per well, 5×10^4^ cells per cm^2^) in each of the 24 hour treatment and recovery conditions. Total RNA was isolated from cells in each group (two independent assays with n = 2–3 wells per group) using the RNEasy kit (Qiagen, Valencia, CA, USA). Primers for bovine interleukin-1 β (IL-1β, Accession: NM_174093, Forward: GGAAATGAACCGAGAAGTGG, Reverse: GCCACAGGAATCTTGTTGTCT), aquaporin-1 (Aqp-1, Accession: NM_174702, Forward: CCTCTCTGCCCGACAACTC, Reverse: GAGTCCCGATGATCTCGATG), and GAPDH (housekeeping gene, Accession: NM_001034034, Forward: GCATCGTGGAGGGACTTATG, Reverse: GGGGCCATCCACAGTCTT) were designed with the Universal Probe Library (Roche, Indianapolis, IN, USA). Amplification reactions were performed using Reverse Transcriptase qPCR MasterMix (Eurogentec, Fremont, CA, USA) and 7900 HT Fast Real-Time PCR System (Applied Biosystems, Foster City, CA, USA). Fold changes in gene expression are reported using the ddCt method [30] relative to respective control groups at each time point (untreated or untreated recovery).

### Immunoblotting

Aqp-1 levels in cell lysates were measured using immunoblotting. NP cells (n = 3 wells per group) were trypsinized, rinsed, and lysed in lysis buffer (50 mM Tris, 150 mM NaCl, 2 mM EDTA, 0.5% triton-X, protease inhibitor, pH 7.5) for 15 mins on ice. Lysates were then sonicated twice for 30 seconds each, followed by incubation at 4°C on a rotator for 3 hours. Lysates were centrifuged at 12500 rpm at 4°C, and supernatants were collected. Total protein levels were measured using BCA assay according to manufacturer’s protocol (Pierce Biotechnology, Rockford, IL, USA). Immunoblotting of lysates was performed using the NuPAGE system (Invitrogen), to detect the presence of Aquaporin-1 (Aqp1) with GAPDH as a loading control. The extracted protein was diluted in 1∶1 in Laemmli buffer containing 10% β-mercaptoethanol, and denatured for 10 min at 70°C. Protein samples (20 ug) were loaded on a 10%, 1.5 mm Bis-Tris gel, separated by electrophoresis (200 V for 50 minutes), and transferred to a PVDF membrane (Millipore, 30 V for 90 minutes). The membranes were blocked for 1 hour in buffer containing non-mammalian Odyssey blocker (927-40100, LI-COR Biosciences, Lincoln, NE), 0.1% Tween and diluted 1∶1 in PBS. Membranes were then incubated with primary antibodies (rabbit polyclonal anti-Aqp1 (1∶400, AB3272, Millipore, Billerica, MA), mouse monoclonal anti-GAPDH (1∶5000, G8795, Sigma Aldrich, St. Louis, MO) in blocking buffer overnight at 4°C. Membranes were washed in the blocking buffer and incubated with secondary antibodies conjugated with infrared dyes (1∶15000, LI-COR Biosciences). Blots were imaged with the Odyssey Imager (LI-COR Biosciences) and densitometry analysis was performed on Aqp-1 and GAPDH bands for each sample. Aqp-1 levels were normalized to GAPDH, and are reported as average ± standard deviation. Specificity of Aqp-1 blotting was confirmed in preliminary experiments, where the presence of protein bands at 28 kDa was no longer visible when samples were co-incubated with the blocking antigen peptide.

### Immunohistochemistry (IHC)

Expression of cell cytoskeletal elements (F-actin and β-tubulin) and of the water channel aquaporin-1 was analyzed. Cells from each treatment and recovery group were plated on poly-lysine coated coverslips and allowed to adhere for 30 minutes (to maintain round morphology as in osmotic loading experiments). For F-actin staining, cells were fixed in 4% paraformaldehyde, permeabilized for 5 minutes with 1% Triton-X 100, and incubated for 30 minutes at room temperature with rhodamine phalloidin (1∶100 dilution, Molecular Probes, Eugene, OR, USA). For β-tubulin staining, cells were fixed in 0.3% glutaraldehyde, permeabilized for 15 minutes with 1% Trition-X 100, and incubated for 90 minutes at 37°C with a mouse anti-β-tubulin primary antibody (1∶200 dilution, Sigma-Aldrich, St. Louis, MO, USA) followed by incubation with Alexa Fluor 488 goat anti-mouse secondary antibody for 60 minutes at 37°C (1∶200 dilution, Molecular Probes, Eugene, OR, USA). Cells were imaged with confocal microscopy (Fluoview 300, Olympus, Center Valley, PA, USA) at 60x magnification and optical slices were taken at 2 µm intervals throughout the cross section. Images of cytoskeletal structure captured at the cell’s midplane are presented.

For aquaporin-1 expression, cells were fixed in 4% paraformaldehyde, permeabilized with 0.5% Triton-X 100, and incubated overnight at 4°C with a rabbit anti-aquaporin-1 primary antibody (1∶200 dilution, Abcam, Cambridge, MA, USA) followed by incubation with an Alexa Fluor 488 goat anti-rabbit secondary antibody for 60 minutes at room temperature (1∶200 dilution, Molecular Probes, Eugene, OR, USA). Cells were imaged via fluorescence microscope (Axiovert 200 M, Zeiss, Thornwood, NY, USA) at 60x magnification using constant exposure time for each image. Quantification of Aqp-1 expression was performed by analyzing cells in ImageJ software, where the mean pixel intensity per cell was computed (n = 35–50 cells per treatment group).

### Statistics

All data is presented as mean ± standard deviation, unless otherwise noted. Nitrite release, gene expression of IL-1β and aquaporin, and cell size were analyzed with two-way ANOVA and Fisher LSD post-hoc test. Intracellular water content and hydraulic permeability were compared with repeated measures ANOVA and Fisher LSD post-hoc test with treatment and osmolarity step as variables. Linear regression analysis was performed between 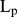 and cell radius in each treatment group. Regression coefficient (R) and p-statistics from the regression analysis are reported. All analysis were performed with STATISTICA software (StatSoft, Tulsa, OK, USA), with p<0.05 considered significant.

## Results

**Figure 1:**
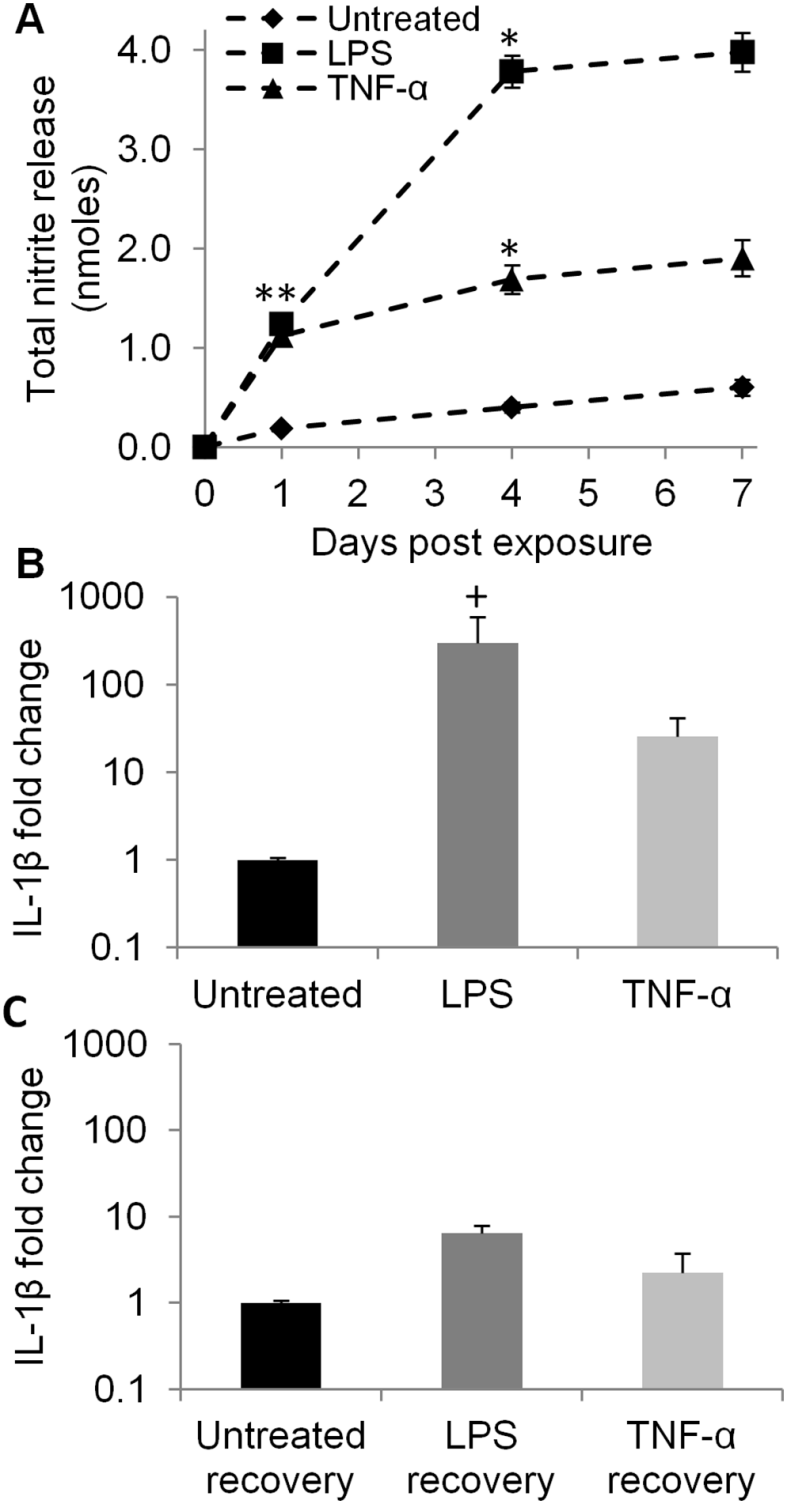
Effects of LPS and TNF-α on inflammatory expression of NP cells. (A) Total nitrite release at days 0, 1, 4, and 7 post stimulation. *p<0.001 relative to the immediately preceding time point. (B) IL-1β gene expression level measured after 24 hours (untreated, LPS, TNF-α) and (C) after 1 week recovery period (untreated recovery, LPS recovery, TNF-α recovery). IL-1β gene expression levels in B and C are normalized to untreated or untreated recovery groups, respectively. ^+^p<0.001 vs. untreated or untreated recovery group.

Treatment of NP cells with LPS or TNF-α for 24 hours resulted in significant increases in inflammatory signaling (Figure 1). Nitrite production was significantly increased in both the LPS (1.1±0.1 nmoles) and TNF-α (1.2±0.1 nmoles) treatment groups at 24 hours versus untreated controls (0.1±0.02 nmoles) (Figure 1A). After 24 hours the cells were returned to growth media, but nitrite release in treated groups continued to increase over control group up to day 4. Between days 4 and 7 the nitrite release rate for treated cells returned to control levels. Treatment with LPS for 24 hours significantly elevated IL-1β gene expression by 532 fold. A 31 fold increase in IL-1β expression due to TNF-α treatment was observed, though it was not statistically significant (Figure 1B). By day 7, IL-1β expression in both the LPS and TNF-α recovery groups was not found to be significantly different from untreated recovery controls.

**Figure 2:**
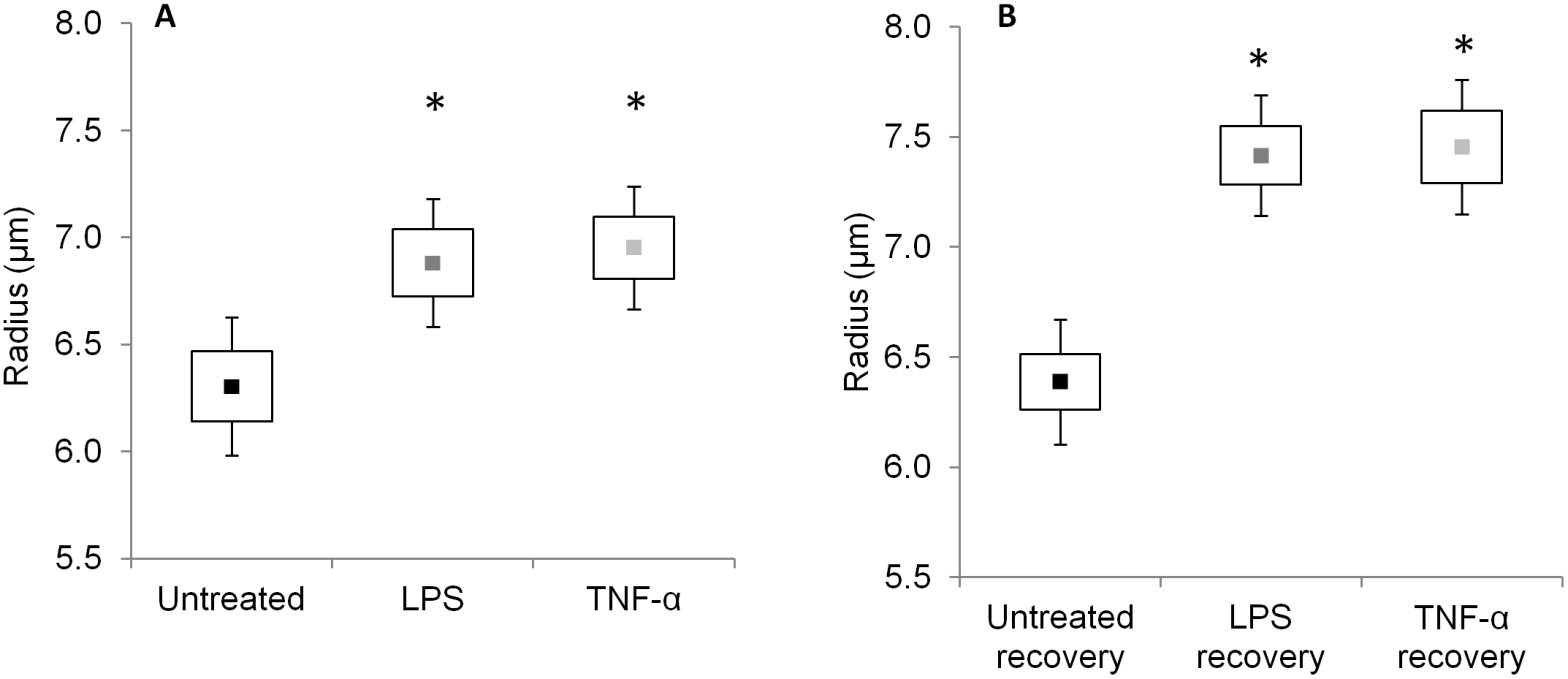
Cell radius measured at 333 mOsm/L at (A) 24 hour and (B) 7-day recovery time points. Data shown is mean (▪), standard error (boxes), and 95% confidence intervals (whiskers; n = 52–127 cells per group). *p<0.03 vs. untreated or untreated recovery control.

Inflammatory treatment was found to irreversibly modulate cell size of NP cells at baseline osmolarity (333 mOsm/L, Figure 2). Cell radius measured at 333 mOsm/L was significantly higher in LPS (6.9±1.1 µm, p<0.03) and TNF-α (7.0±1.3 µm, p<0.01) groups relative to untreated controls (6.3±1.2 µm; Figure 2A). These changes persisted after the recovery period, where significantly greater radii were measured in LPS recovery (7.4±1.4 µm, p<0.001) and TNF-α recovery (7.5±1.6 µm, p<0.001) cells relative to untreated recovery group (6.4±1.4 µm; Figure 2B). No significant change in cell radii was observed between untreated and untreated recovery groups (Figure 2A, B).

**Figure 3:**
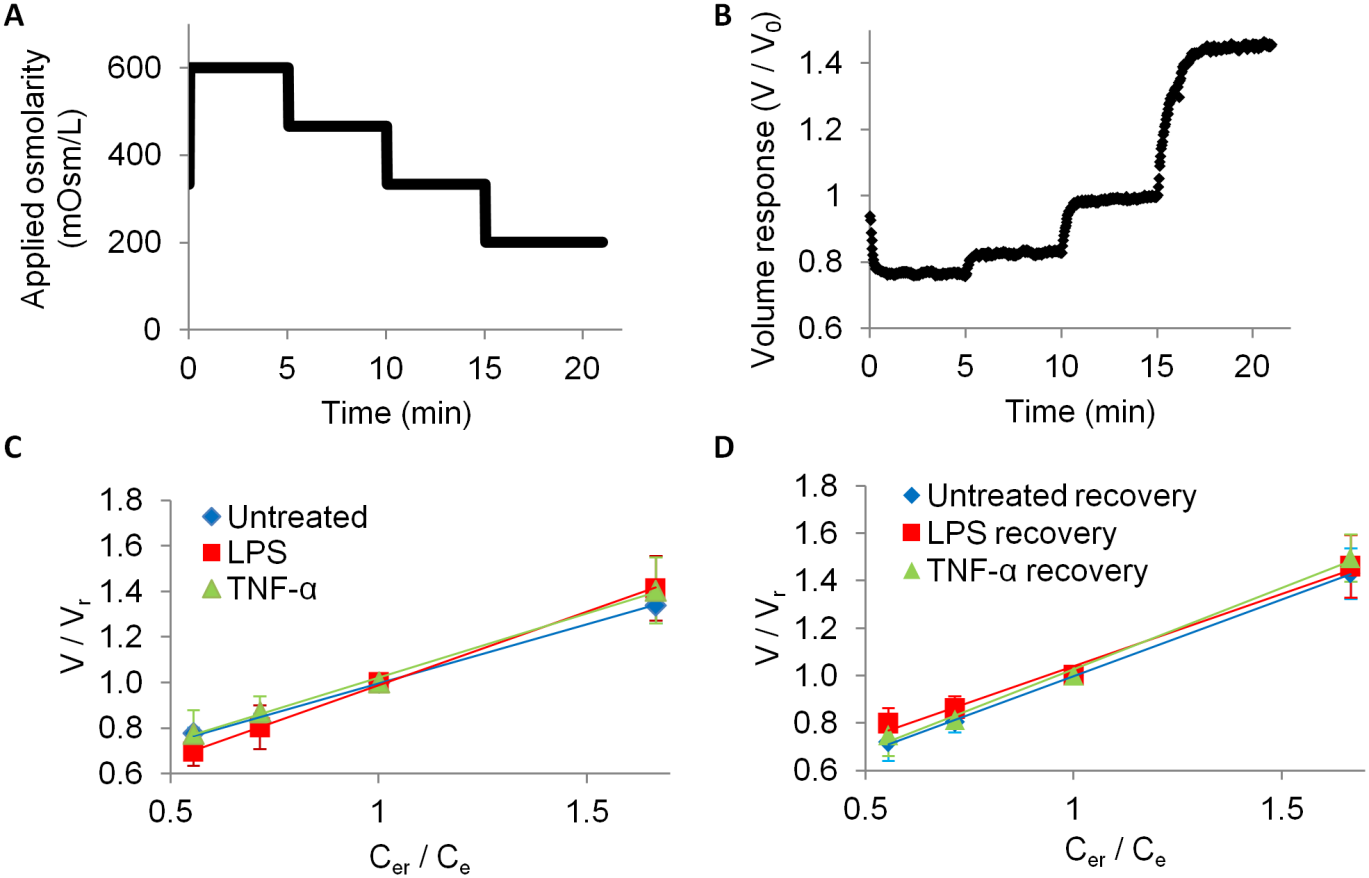
Osmotic loading regime, representative volume-response curve, and equilibrium volume-concentration responses. (A) Osmolarity was applied in a step-wise fashion using decreasing concentrations of NaCl solutions. Each osmotic load was maintained for approximately 5 minutes to allow the cell volume change to reach equilibrium. (B) Representative time-dependent volume of an untreated cell. (C, D) Equilibrium volume (V), normalized by reference volume (V_r_), is plotted as a function of applied external reference osmolarity (c_er_) over current external osmolarity (c_e_) for cells at (C) 24 hour and (D) 7-day recovery time points (n = 7–12 cells per group). The reference osmolarity and associated volume were taken at 333 mOsm/L equilibrium.

To test the effects of inflammation on biomechanical properties, NP cells from each of the treatment and control groups were osmotically loaded under step changes in NaCl solutions of decreasing osmolarity, where the cells were first exposed to a hyperosmotic load (600 mOsm/L), followed by step decreases in osmolarity (466, 333, and 200 mOsm/L; Figure 3A). This caused the cells to initially shrink and then swell monotonically as the osmolarity was lowered (Figure 3B). During each step, images were acquired at a frequency of 0.5 Hz over the duration of 5 minutes, which was sufficient to allow cells to reach equilibrium volume. No regulatory volume recovery was observed in any of the groups. At equilibrium, the relative volume change (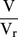) increased linearly with decreasing relative external osmolarity (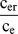), indicative of the Boyle-van’t Hoff relation for a perfect osmometer (Figure 3C). NP cells in control and treatment groups exhibited the behavior of a perfect osmometer (R^2^ = 0.99 for all groups), and no significant differences in equilibrium volume changes were observed among the groups either at 24 hour or 1 week recovery time point (Figure 3C, D).

**Figure 4:**
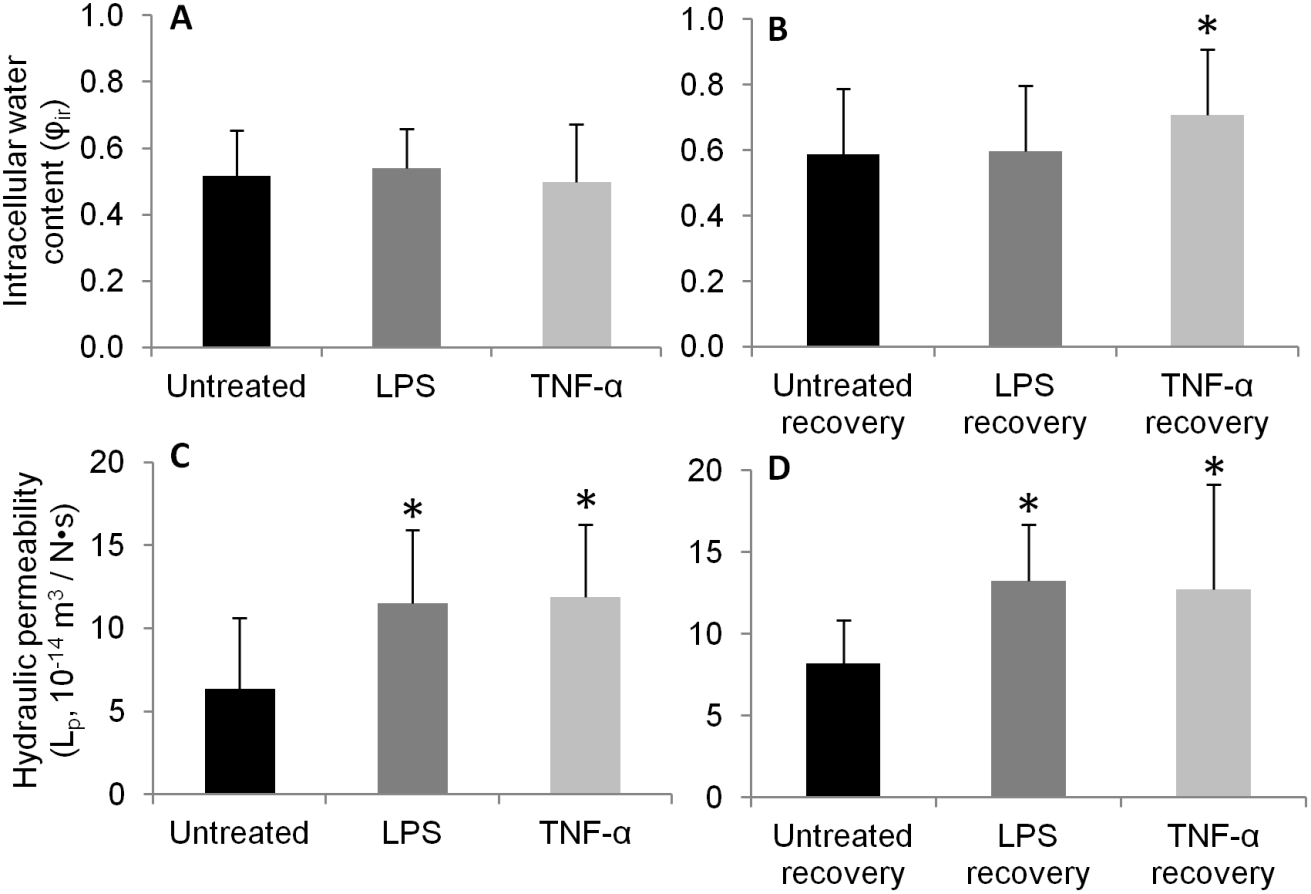
Mean intracellular water content (A, B) and mean hydraulic permeability (C, D) at 333 mOsm/L step for cells at (A, C) 24 hour and (B, D) 7-day recovery time points, respectively. *p<0.05 vs. untreated or untreated recovery control. n = 7–12 cells per condition.

Intracellular water content (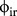) increased monotonically as cells were exposed to decreasing osmolarity steps (Table 1). Significant increase in 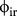 were observed at the most hypotonic step (333 to 200 mOsm/L) relative to 466 to 300 mOsm/L step for cells from TNF-α group, and all groups at recovery time points (Table 1). Treatment with LPS or TNF-α for 24 hours had no significant effect on 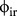 relative to untreated cells, and only the TNF-α recovery group had a significantly greater 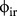 relative to untreated recovery group (Figure 4A, B; p<0.05). Cell hydraulic permeability (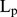) are presented in table 2. Significant increases in 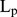 were observed at the most hypotonic step (333 to 200 mOsm/L) relative to 466 to 300 mOsm/L step for cells in the LPS, TNF-α and TNF-α recovery groups (Table 2, p<0.05). Significant increases in 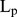 were observed after 24 hours of LPS or TNF-α treatment relative to untreated control (Figure 4C; p<0.05). Significantly increased 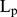 was also observed in LPS and TNF-α recovery groups versus untreated recovery group (Figure 4D; p<0.03). The week-long culture duration did not significantly alter 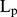 in untreated cells (p>0.33, Figure 4C, D). Overall, curve-fitting of the transient cell response to osmotic loading with the mixture-theory model resulted in a high mean coefficient of determination (R^2^ = 0.87±0.17).

**Table 1:** Intracellular water content determined by curve-fiting the volume-response curves at each osmolarity step.

Intracellular water content ( 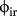 )			
	Osmolarity step (mOsm/L)		
Treatment	600 to 466	466 to 333	333 to 200
Untreated	0.41±0.16	0.52±0.14	0.54±0.15
LPS	0.45±0.22†	0.54±0.12	0.62±0.20
TNF-α	0.43±0.17	0.50±0.17	0.61±0.20†
Untreated recovery	0.49±0.18	0.59±0.15	0.63±0.16†
LPS recovery	0.43±0.13†	0.60±0.18	0.70±0.20†
TNF-α recovery	0.48±0.14†	0.71±0.13*	0.91±0.27* †

Mean ± standard deviation,

*p<0.05 vs. untreated or untreated recovery control;

† p<0.05 vs. 466 to 333 mOsm/L step.

**Table 2:** Hydraulic permeability values generated by curve-fitting the volume-response curves at each osmolarity step.

Hydraulic permeability 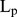 (10^−14^ m^3^/N•s)			
	Osmolarity step (mOsm/L)		
Treatment	600 to 466	466 to 333	333 to 200
Untreated	5.28±1.91	7.44±3.91	8.06±3.01
LPS	9.81±3.83	12.42±4.40*	14.05±4.05* †
TNF-α	9.13±4.83	11.88±4.38*	14.57±5.61* †
Untreated recovery	7.00±2.85	8.18±2.67	8.71±2.87
LPS recovery	10.08±3.35†	13.23±3.44*	14.38±5.69*
TNF-α recovery	12.64±6.09*	12.72±6.44*	15.84±9.01* †

Mean ± standard deviation,

*p<0.05 vs. untreated or untreated recovery control;

† p<0.05 vs. 466 to 333 mOsm/L step.

**Figure 5:**
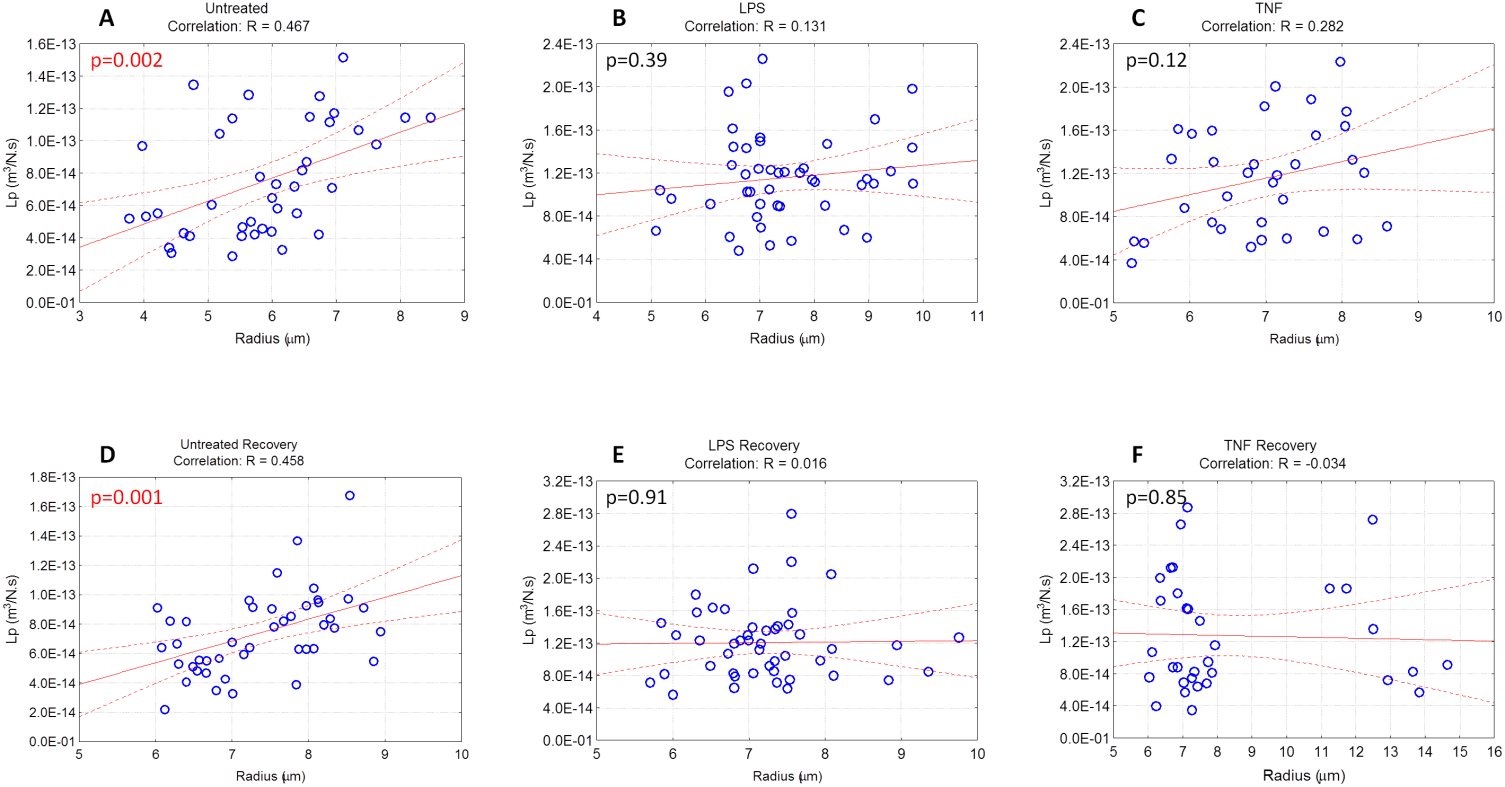
Regression analysis between 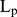 and cell radius in each treatment group. Significant linear correlations were observed for cells in (A) untreated and (D) untreated recovery groups (p<0.05, indicated in red). No significant correlations were observed for cells from LPS or TNF-α at 24 hours (B, C) or at recovery time points (E, F). ‘R’ represents the regression coefficient for each group.

Using regression analysis, the relationship between cell radius and hydraulic permeability were examined in all treatment groups (Figure 5). A significant linear correlation was observed between 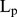 and radius in cells from untreated control group (p<0.002, R = 0.467; Figure 5A). A similar linear correlation was also observed for cells in untreated recovery group (p<0.001, R = 0.458; Figure 5D). In LPS and TNF-α treated groups, no significant linear relationships were observed between 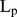 and radius (R = 0.131 and 0.282, respectively; p>0.1; Figure 5B, C). Similarly, cells in LPS recovery and TNF-α recovery groups did not exhibit linear correlations between 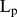 and radius (Figure 5E, F; p>0.8).

**Figure 6:**
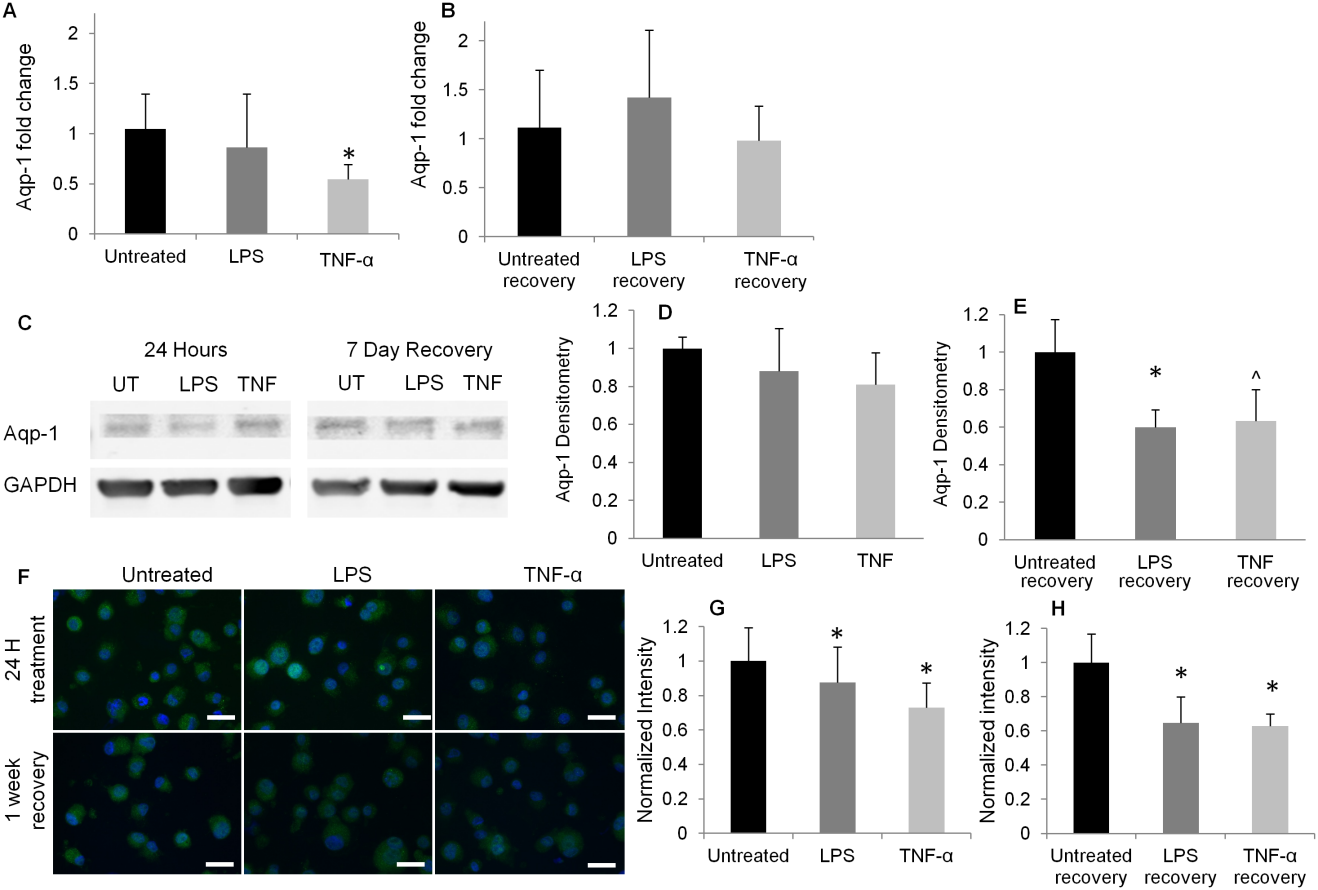
Aquaporin-1 expression in NP cells. (A, B) Gene expression of aquaporin-1 at 24 hour or 7-day recovery time points. Expression levels are normalized to untreated time point control groups (*p<0.05 vs. control). (C) Representative immunoblot of Aqp-1 and (D, E) densitometry of Aqp-1 in NP cells at 24 hour or 7-day recovery time points, respectively. Expression levels are normalized to untreated time point control groups. *p<0.05 vs. time point control, ∧p = 0.07 vs. time point control. (F) Representative immunohistochemical (IHC) staining of Aqp-1 (green) and cell nuclei (blue, scale bars = 20 µm). (G, H) Quantification of Aqp-1 expression measured by IHC, normalized to time point untreated control group (*p<0.05 vs. time point control).

Effect of inflammatory stimulation on the expression of the water channel Aqp-1 was examined (Figure 6). Gene expression of Aqp-1 was found to be similar in LPS treated group relative to untreated control cells, both at 24 hour and at 1 week recovery time points (Figure 6A, B). Aqp-1 gene expression in the TNF-α treatment group was found to be significantly lower relative to untreated control at the 24 hour time point (p<0.04, Figure 6A), though not at the 1 week recovery time point (Figure 6B). Immunoblotting analysis of Aqp-1 and associated densitometry quantification are presented in Figure 6C–E. Aqp-1 protein expression was comparable in cells from control, LPS and TNF-α groups at 24 hours (Figure 6D). At recovery time points, Aqp-1 expression in LPS recovery group was significantly less than in untreated recovery group (p<0.05, Figure 6E). A trend for decreased Aqp-1 expression was also observed between TNF-α recovery and untreated recovery groups (p = 0.07, Figure 6E). Cellular analysis of Aqp-1 with IHC indicated that Aqp-1 was constitutively expressed in NP cells in untreated control groups (Figure 6F). Aqp-1 expression significantly decreased by 13%±3% in the LPS treated group and by 27%±5% in the TNF-α treated group relative to untreated control (p<0.001, Figure 6G). Similarly, decreases in Aqp-1 expression were observed in LPS recovery (35%±8%) and TNF-α recovery (37%±4%) groups relative to untreated recovery (p<0.001, Figure 6H). No significant differences in Aqp-1 were found for untreated cells between the 24 hour and 1 week time points, but LPS and TNF-α treated cells had significantly reduced Aqp-1 expression at the recovery time point as compared with 24 hour treatment (p<0.01).

**Figure 7:**
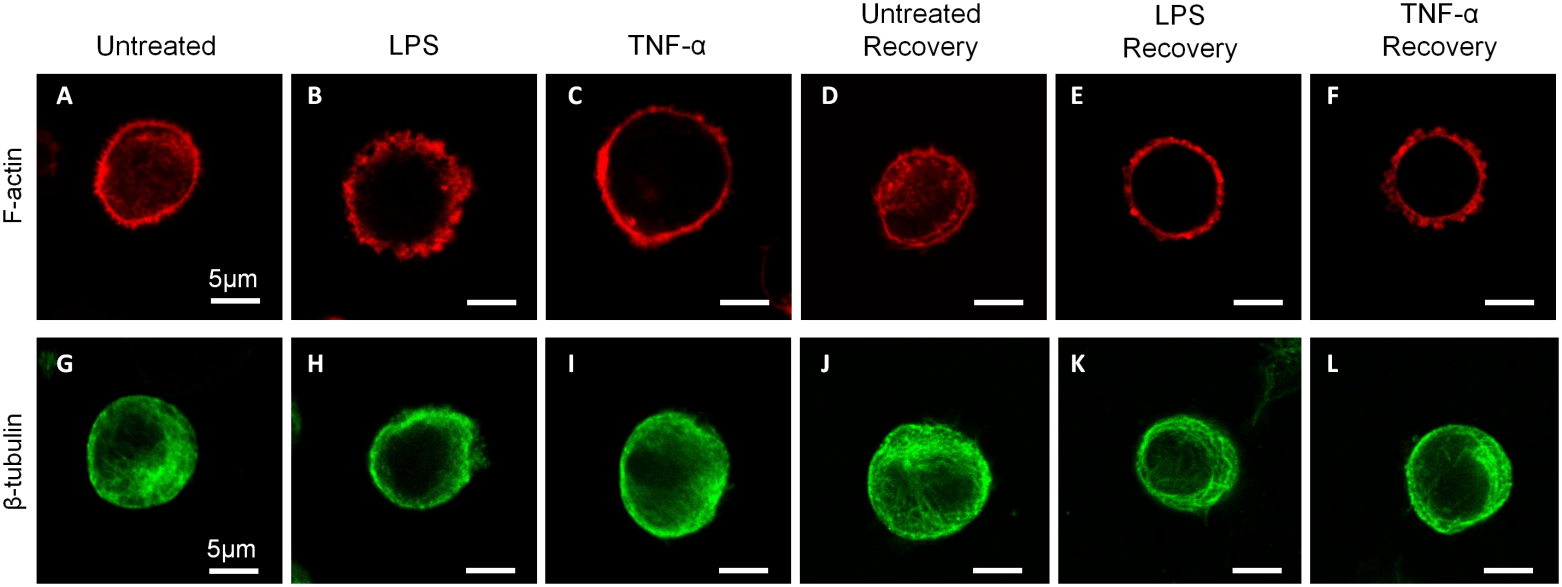
Cytoskeletal structure of rounded NP cells. Representative cytoskeletal staining of NP cells in each treatment group for (A–F) F-actin (red) and (G–L) β-tubulin (green), scale bar = 5 µm. Confocal z-stack images were acquired with 2 µm spacing throughout cell cross-sections, and images represent the slice acquired nearest the center of the cell (midplane).

Cytoskeletal organization of actin and tubulin structures in NP cells are presented in Figure 7. Confocal imaging of NP cells in rounded morphology demonstrated differences in the cytoskeletal structure in LPS and TNF-α treated cells relative to untreated controls (Figure 7). In untreated cells, F-actin was observed in the cell midplane as a fibrous network throughout the cytoplasm and as a dense structure outlining the cortical periphery of the cell (Figure 7A). In LPS and and TNF-α treated cells at 24 hours, actin structure was found to be disrupted relative to untreated cells as exhibited by decreased presence of cytoplasmic actin network (Figure 7B, C). Expression of cortical actin along the cell periphery was observed in LPS and TNF-α treated cells. Similar alterations in F-actin distribution were observed in the LPS and TNF-α recovery groups relative to untreated recovery (Figure 7 D–F). β-tubulin distribution was also found to be moderately altered in the inflammatory stimulated groups relative to untreated controls, though differences were less distinct than those seen for F-actin (Figure 7G–L). β-tubulin expression was slightly diminished in inflammatory groups relative to their untreated time point controls.

## Discussion

The goal of this study was to measure the effect of inflammatory stimuli on the response of NP cells to osmotic loading. Our findings demonstrate that inflammation causes biomechanical and structural changes in NP cells including increased hydraulic permeability and cell size. These changes persist after removal of the inflammatory stimulus, suggesting that the cells cannot spontaneously recover from inflammatory exposure *in vitro*. It is well established that chronic inflammation in the disc is associated with ECM loss and degradation [15], [17]. The findings of the current study identify biophysical and cytoskeletal effects of inflammation directly on disc cells, which may regulate cell mechanobiology in degeneration and inflammatory disease.

To our knowledge, this is the first study examining cellular-level biomechanical changes in NP cells induced by inflammation. Inflammatory treatment for 24 hours significantly increased the hydraulic permeability and cell radius (Table 2, Figures 2, 4). A regression analysis between 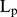 and radius of untreated NP cells at 24 hours or at recovery time points demonstrated a significant linear correlation (Figure 5A, D). Our findings indicate that NP cell hydraulic permeability is size-dependent when cultured without inflammatory stimuli (Figure 5). This relationship is consistent with a previous study demonstrating significant correlations between cell size and viscoelastic properties of healthy porcine NP cells [31]. In our study, changes in 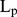 persisted after removal of inflammatory stimuli and cells were cultured in basal media for an additional 1 week recovery; meanwhile, the production of some inflammatory mediators (nitrite and IL-1β) had returned to near-basal levels during the recovery time frame (Figure 1). Moreover, no significant linear correlation was observed between 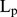 and radius for cells treated with LPS or TNF-α after 24 hours or 1 week recovery. This loss of correlation between cell size and hydraulic permeability suggests that regulation of volume change is disrupted irreversibly due to inflammatory stimulation. Brief exposure to inflammation may have significant consequences on cell biomechanical properties, regardless of the recovery microenvironment to non-inflammatory levels, *in vitro*. In this study, we chose to measure the gene expression of inflammatory cytokine IL-1β as a mediator of the inflammatory stimulation experiment. IL-1β has been shown to be upregulated in NP cells during disc degeneration *in vivo* [17], [20] and upregulated in response to LPS or TNF-α treatment *in vitro* [23].

The influence of inflammation on the osmotic behavior of NP cells at a wide osmotic range mimicking the *in situ* osmolarity of the intervertebral disc during physiological activity was examined [7], where osmotic loads in the range of 450–550 mOsm/L or 100–200 mOsm/L have been reported in normal vs. degenerated discs, respectively [2], [14]. Step osmotic loading resulted in transient cell-volume responses followed by sustained equilibrium (Figure 3B). The steady-state volume response of cells in each treatment group demonstrated a linear behavior over the applied osmolarities (Figure 3C, D) consistent with the Boyle-van’t Hoff relation for a perfect osmometer [32] which is expected when regulatory volume changes are negligible. Loading with solutions at physiologic temperature and composition could help elucidate the effects of inflammation on active cellular response in future studies. Untreated NP cells exhibited an average hydraulic permeability (Table 2) similar to that previously reported for articular chondrocytes, but the intracellular water content was lower than previously reported in chondrocytes (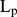 = 6.84±2.23×10^−14^ m^3^/Ns, 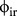 = 0.73±0.06) [27].

Cellular expression of Aqp-1, the water channel isoform reported to be most abundant in the human IVD [33], was found to decrease with inflammatory stimulation in NP cells (Figure 6G, H). Consistent with our findings, Aqp-1 has been shown to be downregulated in murine epithelial cells in response to inflammatory treatment by LPS [34]. Overall, increases in Aqp-1 expression are associated with increases in membrane water channels, which can accelerate the cell swelling rate by providing a greater number of conduits for water transport [35]. Aqp-1 expression at the cellular level (indicated by IHC) was significantly lower in LPS and TNF-α treated and recovery groups compared to untreated controls (Figure 6G–H). These findings suggest that the observed permeability differences in inflammatory-stimulated cells are not directly correlated with Aqp-1 cellular expression in NP cells. Alternatively, other aquaporin isoforms, not investigated in the current study, may be contributing to the permeability increases. Interestingly, we did not observe significant differences in Apq-1 gene expression between untreated and LPS treated cells at both time points, suggesting that inflammatory stimulation regulates Aqp-1 transcription more than gene expression in NP cells. We analyzed and quantified Aqp-1 expression using IHC on a large number of cells (n = 35–50 cells per treatment group) to statistically confirm the decreases in Aqp-1 expression due to inflammation. Immunoblotting of Aqp-1 demonstrated a trend for decreased expression with inflammation at both time points. However, the relatively small effect size expected (∼10–20% difference between groups) was not statistically confirmed with immunoblotting due to the limited number of samples (n = 3) available using this technique. Previous studies have shown that Aqp-1 suppression decreases cell adhesion and migration in murine chondrocytes [36] and human endothelial cells [37] by inducing re-organization of F-actin. Therefore, our observations on alterations in F-actin in inflammatory stimulated NP cells may be related to Aqp-1 levels. Increased cell size and hydraulic permeability due to inflammation appear to be predominantly mediated by cytoskeletal disruption, with changes in Aqp-1 expression potentially contributing compensatory factors or secondary effects to the hydraulic permeability changes. Interestingly in joint diseases associated with inflammation, it has been shown that Aqp-1 is upregulated in synoviocytes from patients with rheumatoid arthritis [38] and fibrochondrocytes from osteoarthritic joints [39]. While inflammation may be one component of the disease etiology, it is unknown in these clinical models whether the changes in Aqp-1 expression are induced by the inflammatory or other mediators of the disease.

We observed significant differences in cytoskeletal structure for LPS and TNF-α treated cells, especially in F-actin organization, relative to untreated controls (Figure 7). In the current study, actin was expressed throughout the cytoplasm and at the cortex of untreated NP cells, whereas expression in inflammatory treated NP cells was observed primarily at the cell cortex. This disruption in actin expression remained after the recovery period in inflammatory treated cells relative to untreated recovery. We have also observed actin structure in flattened NP cells and found expression to be dominated by elongated filaments, whereas inflammatory treatment of flattened NP cells resulted in a disrupted, puctate actin distribution (data not shown), suggesting that inflammation disrupts actin polymerization in NP cells. Our findings are consistent with previous reports on the effects of inflammatory stimulation on actin structure. In articular chondrocytes, treatment of cells with exogenous nitric oxide (an inflammatory mediator) was found to disrupt elongated actin filaments into short, rod-like, randomly arranged structures [40]. In fibroblasts, TNF-α exposure was shown to disrupt the parallel array of stress fibers normally observed in the perinuclear region [41].

The cytoskeleton contributes to the biomechanical properties of the NP cell, which also influences the interactions between the cell and its pericellular and extracellular matrix. Disruption of actin microfilaments by cytochalasin D treatment was shown to significantly reduce the elastic moduli and apparent viscosity in NP cells and resulted in a faster hyperosmotic stress response compared to untreated cells [42]. Since cell membrane regulation is linked to underlying cytoskeletal structure [43], our observed biophysical and cytoskeletal differences may be related to the regulation of cell membrane folding. Indeed our observation that hydraulic permeability and cell size are correlated in untreated cells supports the notion that cell swelling increases hydraulic permeability, possibly by unfolding the cell membrane. In the presence of inflammatory stimulation, we theorize that inflammatory mediators act as triggers for disruption of the actin cytoskeleton (Figure 7), resulting in loss of cellular pre-stress and increased cell size (Figure 2). Increases in cell size due to inflammation may subsequently induce some unfolding of the cell membrane, resulting in increased hydraulic permeability (Figure 4) without significant increases in Aqp-1 required (Figure 6). The cytoskeletal disruption findings (Figure 7) and the loss of linear correlation between 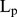 and radius in inflammatory stimulated cells at both time points (Figure 5) further confirm that these changes are irreversible in the current model system. Further studies are needed to determine if cell cytoskeletal changes are causative of changes in osmotic biophysical properties. The potential role of the cytoskeleton in the degenerative cascade associated with disc matrix catabolism, and the relationship between Aqp-1 and F-actin structure remain to be elucidated.

A potential limitation of the current study is that the use of 2D culture may alter the NP cell phenotype [44], and may differentially regulate the inflammatory response [45]. However, NPs are known to respond to cytokines both *in vivo* and *in vitro* [20], and our findings on biophysical responses are assessed on cells in 3D-like rounded morphology. Another limitation of the current study is that the NP cells were examined in an isolated, free-swelling environment. *In vivo*, these cells are surrounded by pericellular and extracellular matrices that confine the cells and contribute to cell volume regulation [46]. Furthermore, these cells actively regulate their volume *in vivo* by increasing or decreasing the intracellular concentration of osmolytes. It has also been suggested that this process is dependent on the F-actin cytoskeleton [42], which we found to be disrupted by the inflammatory stimulation in the current study. The downstream consequences of altered cell mechano-sensitivity, including cell viability, metabolism, and ECM turnover, remain to be elucidated. Future studies will characterize the biophysical responses of NP cells to inflammatory stimulation in a 3D microenvironment that accounts for cell-matrix interactions.

In summary, we found that the inflammatory stimuli LPS and TNF-α induced significant changes in the volume-response of NP cells to osmotic loading, including an increase in cell hydraulic permeability and cell size. These changes are associated with alterations in cytoskeletal structure in inflammatory-treated cells. Treated NP cells were unable to recover their biophysical properties to that of untreated cells after removal of inflammatory stimulant and return to basal media for 1 week. This finding suggests that therapeutic treatment beyond recovery from inflammatory signaling may be necessary to stabilize the cytoskeletal structure and to recover the biophysical properties of NP cells. These findings may also be useful in models of cellular behavior and mechanobiology in response to mechano-osmotic loading in healthy and diseased tissues.
